# Supplementary material for: Medial prefrontal cortex Notch1 signalling mediates methamphetamine-induced psychosis via Hes1-dependent suppression of GABAB1 receptor expression
Source: Mol Psychiatry. 2022 Jun 22;27(10):4009–22. doi: 10.1038/s41380-022-01662-z (PMC9718672; doi:10.1038/s41380-022-01662-z)
Supplement: Supplementary file 1 — supplement information [file 41380_2022_1662_MOESM1_ESM.docx]

**Supplemental methods**

**Behavioural Testing**

**Open filed test**

Behavioural effects of the MK-801 treatments were quantified using the open field test. Individual mice were placed in the metal test chambers (43 × 43 × 43 cm) and analyzed the day after last MK-801 injection using a smart 2.5 video tracking system. The total distances moved throughout the area as well as the time spent in the centre zone were recorded for 10 min ^1^.

**Y-maze spontaneous alternation**

The Y-maze was a Y-shaped apparatus with three arms, each 30 cm long and 6 cm wide with walls 15 cm high. The arms were at a 120° angle from each other. Mice were randomly placed at the end of one of the 3 arms to avoid placement bias and allowed to freely explore the Y-maze for 5 minutes. A correct alternation was defined by a trio of arm entries in which the animal accessed all three arms in sequence without repetition (ABC, BCA, and CAB). Alternation triplet = [(correct alternations)/(total arm entries − 2)] × 100% ^2^.

**Novel object recognition task (NOR)**

The procedure for this test follows Antunes et al ^3^. Animals were individually habituated to an empty box (30 × 45 × 16 cm high) for 10 min on two consecutive days. During the training phase, two identical objects (A and A) were placed in the middle area of the chamber. Each animal was then placed in the box and allowed to freely explore the area for 5 min. This trial was repeated three times at 15 min intervals. Three hours after the training phase, one object (A or B randomly) was substituted for a novel object (C), and exploratory behaviour was again evaluated for a total of 5 min in test 1. For the last test, object C was replaced by another novel object (D). During the trials, the open field box and all objects were thoroughly cleansed using 70% ethanol between sessions to prevent odour recognition. Exploration of an object was characterized as sniffing it at a distance of less than 2 cm, and/or touching it with the nose. Smart 3.0 software was used to record the exploration time. Discrimination of visual novelty was assessed by a recognition index, defined as: new object exploration time / (new object exploration time + old object exploration time) × 100%. Animals displaying a dramatic bias for the objects during training (>65% investigation with one object) were excluded from the data pool.

**Elevated plus maze (EPM)**

The EPM consisted of a plus-shaped platform with two open (33 × 6 cm) and two closed arms extending from a 6 × 6 cm central area; the platform was elevated 50 cm off the ground. During the 5-min trial, the time spent in the open arm% and the number of entries into open arm% were measured by Smart 3.0 software ^2^.

**Social interaction test (SIT)**

The social interaction was evaluated using the method previously described by Busquets-Garcia et al ^4^. The testing apparatus consisted of a 43 × 43 cm open field wide with 2 plastic containers (A and B, plastic cylinders of 8 cm diameter with holes for odor interaction) in 2 opposite corners. A chamber contained a probe mouse (8-10 weeks old adult male C57BL/6J), while the B chamber remained empty. Experimental mouse was placed in the middle of the open field for 5 minutes exploration. During this period, social zone was characterized as 8-cm area surrounding the A chamber and non-social” zones was defined as 8-cm area surrounding the B chamber. Smart 3.0 software was used to record and analyse the experimental mouse in different zones. A social interaction index was defined as follows: time spent in the social zone / total time in both zones. After each test session, the observation chamber was cleaned with 70% ethanol to remove residual odor.

**Tail suspension test (TST)**

The TST was performed in accordance with previously described method ^5^. Briefly, the mice were suspended with adhesive tape from a hook 50 cm above soft bedding material in a chamber that was both acoustically and visually isolated. The hook was placed approximately 1 cm from the tip of the tail. The mice were suspended for 6 min, and the immobility duration was recorded and analysed during the last 5 min of the 6 min test using Smart 3.0 software.

**Forced swimming test (FST)**

The FST is performed to assess despair behavior ^5^. Each mouse was placed in a 10-litre transparent plastic cylinder that was filled with tap water at 25 ± 1°C to a depth of 30 cm; the mouse was then left to move freely in the water for 6 min. Immobility was defined as a lack of movements except those necessary to prevent the animal from drowning. The percent immobility was calculated as the percent of immobile behaviour over the test duration. Moreover, the immobility duration was recorded and analysed during the last 5 of the 6 min using Smart 3.0 software. The mouse was immediately taken out of the cylinder and excluded from the study if it failed to swim or keep its head above water.

**References:**

1. Xiu Y, Kong XR, Zhang L, Qiu X, Chao FL, Peng C et al. White matter injuries induced by MK-801 in a mouse model of schizophrenia based on NMDA antagonism. *Anat Rec (Hoboken)* 2014; **297**(8): 1498-1507.
2. Yin F, Guo H, Cui J, Shi Y, Su R, Xie Q, et al. The basolateral amygdala regulation of complex cognitive behaviours in the five-choice serial reaction time task. *Psychopharmacology (Berl)* 2019; **236**:3135-46.
3. Antunes M, Biala G. The novel object recognition memory: neurobiology, test procedure, and its modifications. *Cogn Process* 2012; **13**:93-110.
4. Busquets-Garcia A, Soria-Gómez E, Redon B, Mackenbach Y, Vallée M, Chaouloff F *et al.* Pregnenolone blocks cannabinoid-induced acute psychotic-like states in mice. *Mol Psychiatry* 2017; **22**(11)**:** 1594-1603.
5. Wang J, Lai S, Wang R, Zhou T, Dong N, Zhu L, et al. Dopamine D3 receptor in the nucleus accumbens alleviates neuroinflammation in a mouse model of depressive-like behavior. *Brain Behav Immun* 2021; **101**:165-79.

**Supplemental Table 1**. Summary of forward and reverse primer sequences in quantitative real-time PCR

| Gene | Forward primer sequence (5′-3′) | Reverse primer sequence (5′-3′) |
| --- | --- | --- |
| Notch1 | TCAGGGTGTCTTCCAGATCC | CAGCATCCACATTGTTCACC |
| Jagged1 | TGACATGGATAAACACCAGCA | GCAGCCCACTGTCTGCTATAC |
| RBP-J | CCAATTTCAGGCCACTCCA | TCTACATCCCCAAACCACACTC |
| Hes1 | GCAGACATTCTGGAAATGACTGTGA | GAGTGCGCACCTCGGTGTTA |
| GAT1 | TAACAACAACAGCCCATCCA | GGAGTAACCCTGCTCCATGA |
| GAT3 | CCTCCATGATCTGCATTCCT | CCAAATACCCCCTTTCGTCT |
| GABAAα3 | CAAGAACCTGGGGACTTTCTCAA | AGCCGATCCAAGATTCTAGTGAA |
| GABAA β1 | GGTTTGTTGTGCACACAGCTCC | ATGCTGGCGACATCGATCCGC |
| GABAB1 | ACGTCACCTCGGAAGGTTG | CACAGGCAGGAAATTGATGGC |
| GABAB2 | CCTGGTCATCATCTTCTGTAGCA | AACTGGAATCGCCTGTTCTGA |
| Gapdh | TGTGTCCGTCGTGGATCTGA | TTGCTGTTGAAGTCGCAGGAG |
| Primer1(ChIP) | AGCCTACTGCTGGACTAACGA | AGGGAGAATCAAGGGTCAAAA |
| Primer2(ChIP) | ATACCTGCTTTCCACCCAC | CTCCTGCATTGCGATTGTC |
| Primer3(ChIP) | TTAGTTCGTGGTTTAGGAGTC | GTGGAAAGCAGGTATTATGAG |
| Gapdh | TGTGTCCGTCGTGGATCTGA | TTGCTGTTGAAGTCGCAGGAG |

The relative expressions of these genes were normalized to the Gapdh. The melt temperature (Tm) was kept between 55°C and 65°C.


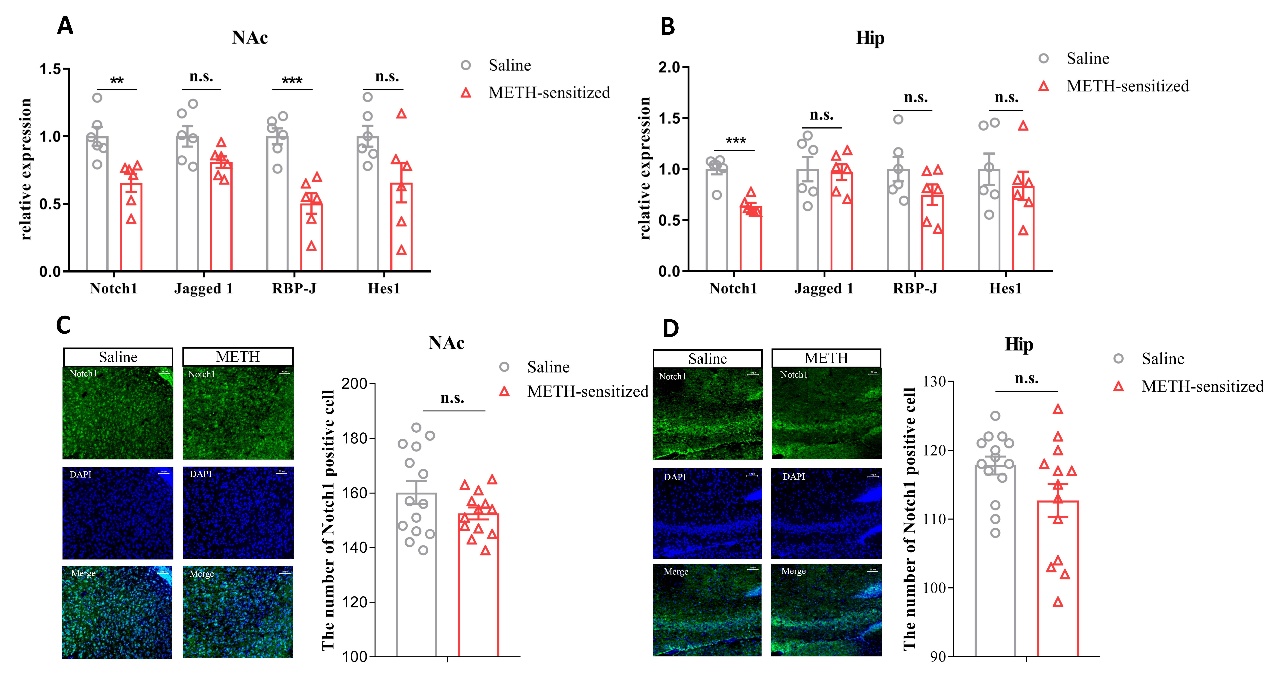


**Supplemental Figure 1. The expression changes of Notch1 signalling in the NAc and Hip of METH-induced sensitized mice. 1A.** mRNA level of the Notch1 signal pathway in the NAc of METH sensitized mice. There was significant downregulation of Notch1 (t_10_ = 3.61, *P* < 0.01) and RBP-J (t_10_ = 5.15, *P* < 0.001) but not Jagged1 (t _10_ = 2.22, *P* > 0.05) or Hes1 (t_10_ = 2.08, *P* > 0.05) by student’s t-test. **1B.** mRNA level of the Notch1 signalling in the Hip of METH-sensitized mice. The Notch1 receptor was significantly reduced (t_10_ = 5.99, *P* < 0.001). However, there were no significant changes of Jagged1 (t_10_ = 0.21, *P* > 0.05), RBP-J (t_10_ = 1.61, *P* > 0.05) or Hes1 (t_10_ = 0.80, *P* > 0.05) by student’s t-test**;** ***P* < 0.01, ****P* < 0.001 vs. saline group. n.s. means no significant changes. Data were presented as mean ± S.E.M, n = 6. **1C-1D.** Representative images of immunofluorescence staining for the Notch1 receptor in NAc (C) and Hip (D) between the METH-sensitized group and the saline group. There was no significant changes of Notch1-positive cells between the two groups in both NAc (C) and Hip (D) by student’s t-test, scale bar = 50 μm, n=3 / group, 10-15 photos. The n.s. means no significant changes. Data were presented as mean ± S.E.M.

**
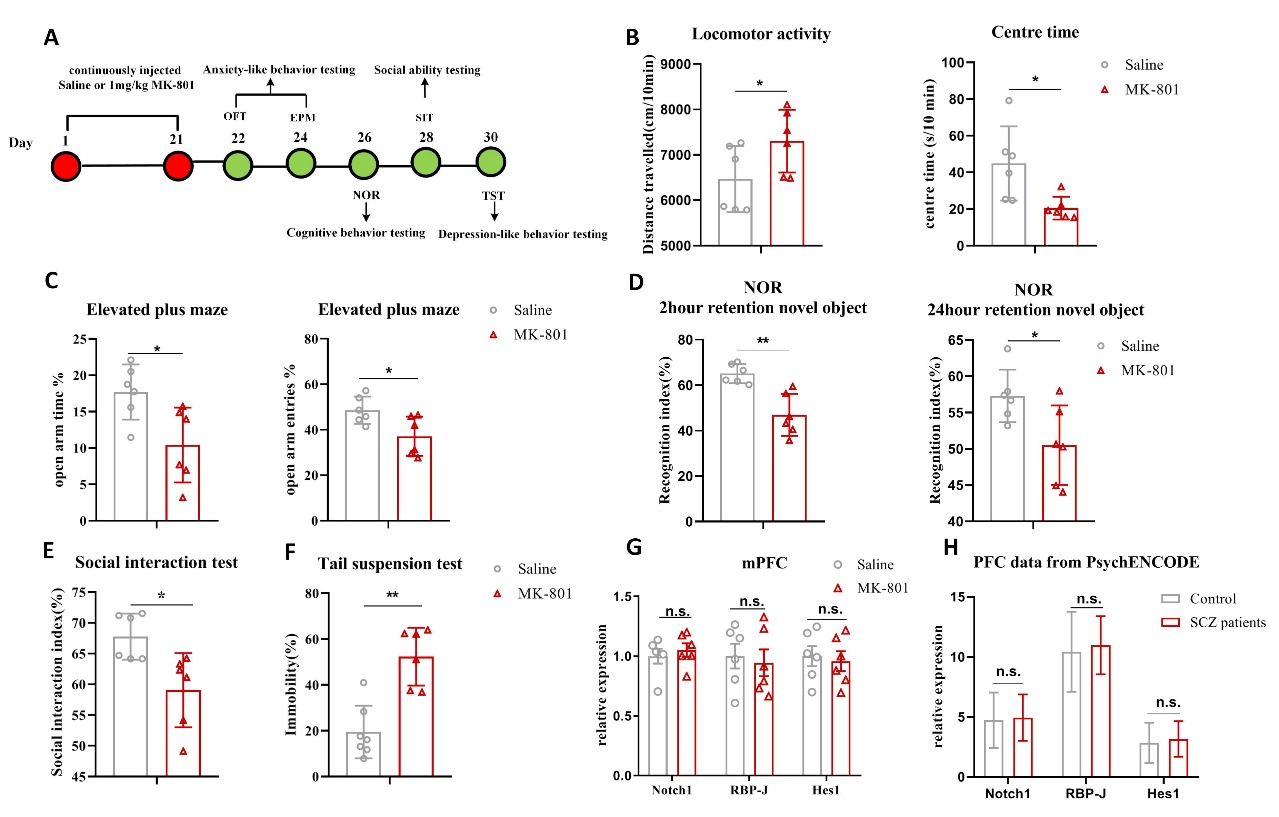
**

**Supplemental Figure 2. The expression changes of Notch1 signalling in the mPFC of MK-801 treated mice. 2A.** Timeline of SCZ relevant behavioural tests in MK-801 treated mice. **2B.** Repeated MK-801 treatment significantly increased locomotor activity (t_10_ = -2.30, *P* < 0.05) and decreased the time spend in centre area (t_10_ = 2.82, *P* < 0.05). **2C.** There were significantly lower proportions of time in the open arms (t_10_ = 2.67, *P* < 0.05) and lower proportions of entries into the open arms (t_10_ = 2.97, *P* < 0.05) of MK-801 treated mice than the control saline group. **2D.** Repeated MK-801 treatment caused significantly reduction in the recognition index (%) after 2 hours (t_10_ =4.38, *P* < 0.01) and 24 hours of training (t_10_ = 2.54, *P* < 0.05). **2E.** The MK-801 group of mice showed a lower social index (%) than the control group (t_10_ =3.00, *P* < 0.05). **2F.** Repeated MK-801 treatment significantly increased percentage of immobility time in the TST (t_11_ =-3.12, *P* < 0.01). **2G.** Changes in the expression of Notch1 signalling components in the mPFC of the MK-801-induced SCZ animal model. There were no significant changes (n.s.) of Notch1 (t_10_ = -0.62), RBP-J (t_10_ = 0.37) or Hes1 (t_10_ = 0.36) in the mPFC of MK-801-treated mice. **2H.** mRNA-sequencing data from the PsychENCODE consortium including 256 healthy people and 95 schizophrenia patients’ prefrontal cortex data. There were no significant changes in Notch1 (t_349_ = -0.80, *P* > 0.05), RBP-J (t_349_ = -1.46, *P* > 0.05) or Hes1 (t_349_ = -1.67, *P* > 0.05) between schizophrenia patients and controls. All these data were tested by student’s t-test. **P* < 0.05, ***P* < 0.01 vs. the saline group. The n.s. means no significant changes. Data were presented as mean ± S.E.M, n = 6-7.


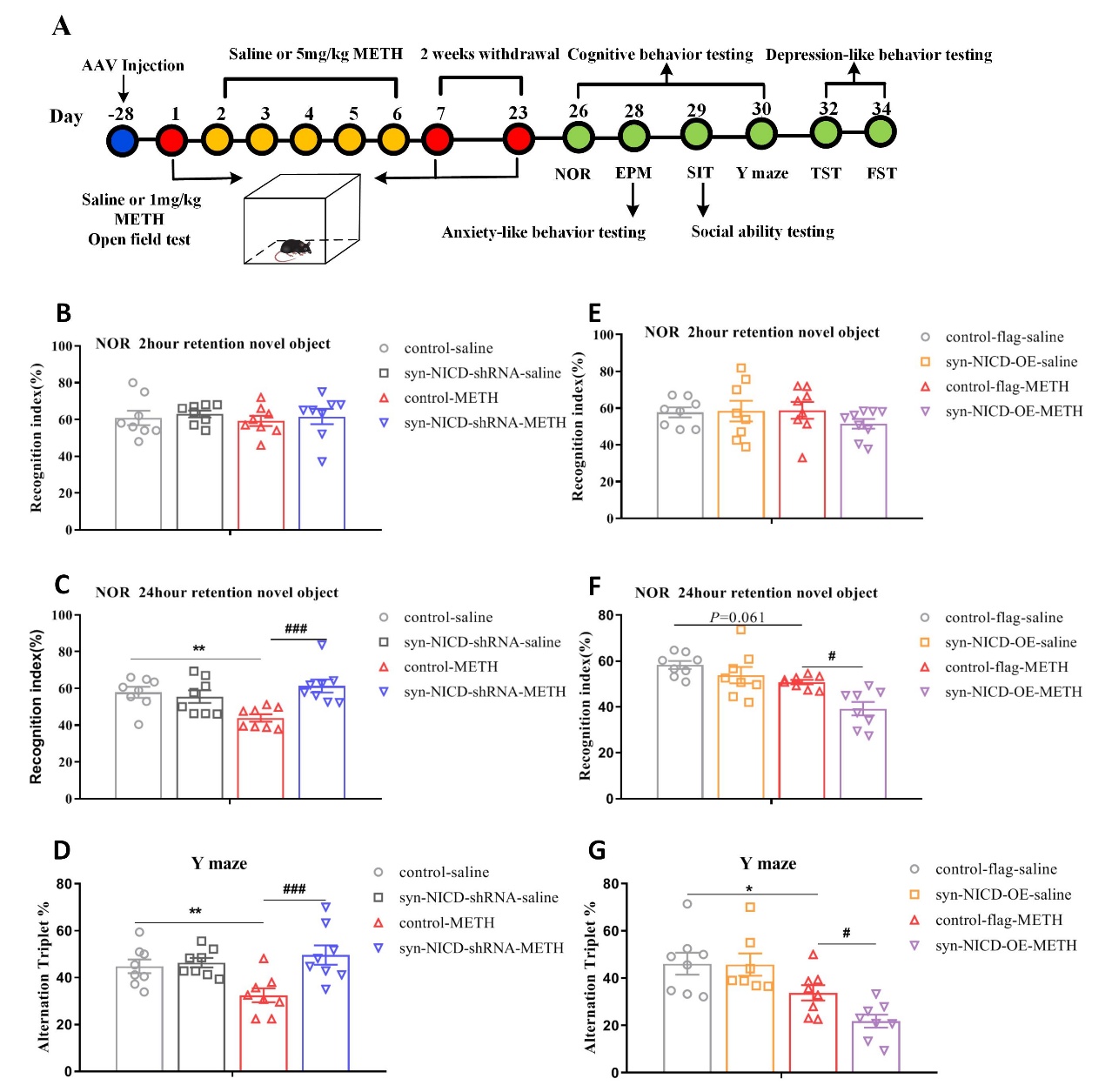


**Supplemental Figure 3.** **Manipulating Notch1 expression affects METH-induced psychosis (NOR and Y-maze). 3A.** Timeline of MIP relevant behavioural test. These behavioural tests were carried out within 2 weeks after the METH challenge injection. **3B-3C.** Inhibition of Notch1 signalling reversed the decrease in the recognition index (%) after 24 hours of training but not after 2 hours. **3C.** Two-way ANOVA with LSD post hoc multiple comparison revealed significant main effect of AAV [F _(1, 28)_ = 6.06, *P* < 0.05] and METH×AAV [F _(1, 28)_ = 10.48, *P* < 0.01], but not the METH (*P* > 0.05). **3D.** Downregulation of Notch1 signalling reversed the reduction in alternation triplet in the Y-maze [main effect of AAV, F _(1, 28)_ = 8.99, *P* < 0.01; METH, F _(1, 28)_ = 2.14, *P* > 0.05; AAV×METH, F _(1, 28)_ = 6.27, *P* < 0.05] **3E-3F.** Overexpression of Notch1 signalling enhanced the decreased recognition index (%) after 24 hours of training but not after two hours. **3F.** Two-way ANOVA with LSD post hoc multiple comparison revealed significant main effect of AAV [F (_1, 28)_ = 10.11, *P* < 0.01] and METH [F _(1, 28)_ = 19.17, *P* < 0.001], but not the AAV×METH (*P* > 0.05). **3G.** Overexpression of Notch1 signalling aggravated the reduction in alternation triplet in the Y-maze [main effect of AAV, F _(1, 27)_ = 2.53, *P* > 0.05; METH, F _(1, 27)_ = 21.84, *P* < 0.001; AAV×METH, F _(1, 27)_ = 2.24, *P* > 0.05].**P* < 0.05, ***P* < 0.01 vs. the saline control group. #*P* < 0.05, ###*P* < 0.001 control-METH vs. syn-NICD-OE-METH or syn-NICD-shRNA-METH group. Data were presented as mean ± S.E.M, n = 7-8.


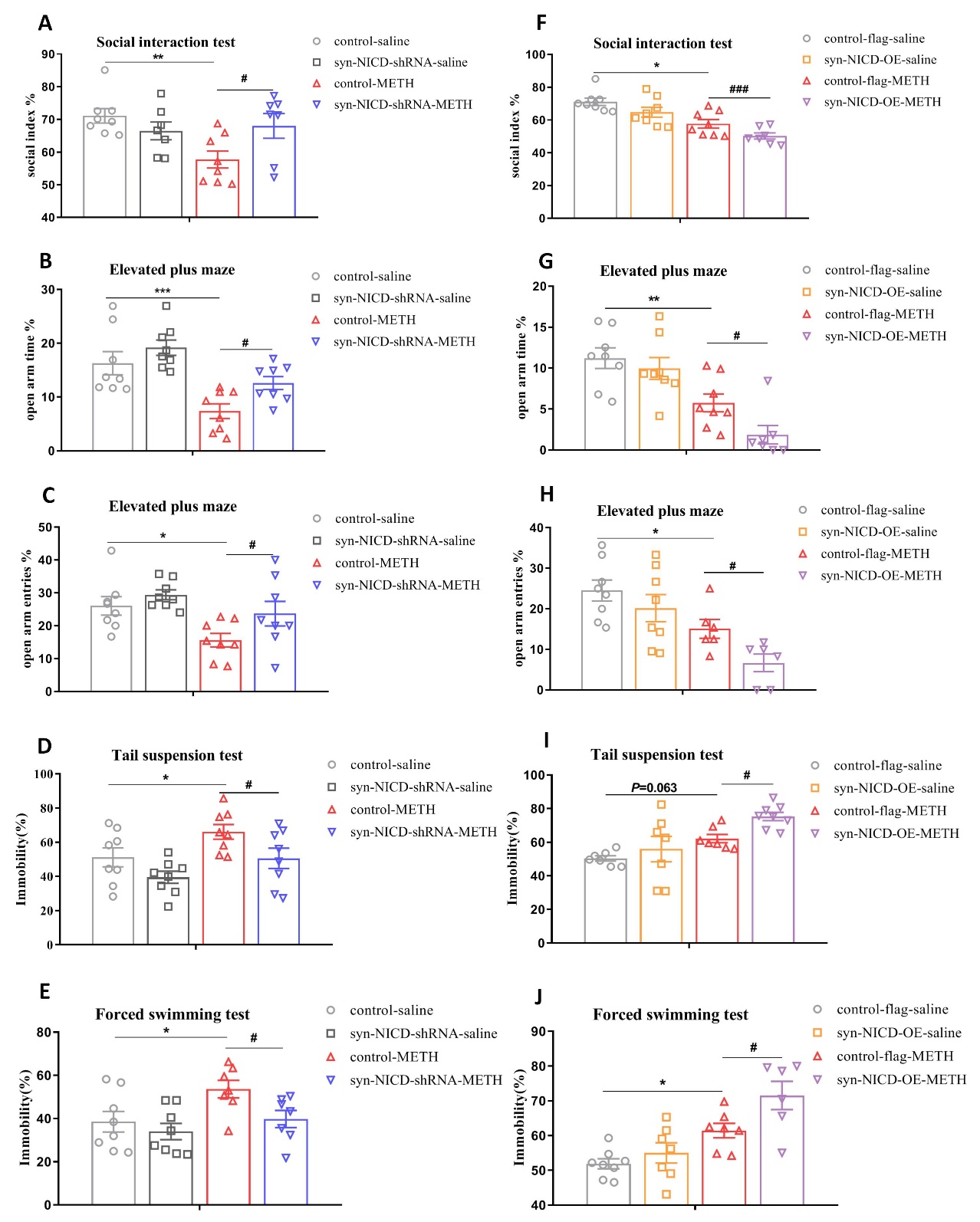


**Supplemental Figure 4. Manipulating Notch1 expression affects METH-induced psychosis (SIT, EPM, TST and FST).** **4A, 4F.** Down-regulation NICD (A) or over-expression NICD (F) in mPFC neurons affected the MIP-associated social interaction changes. **4A**. Two-way ANOVA followed by LSD test showed that the MIP mice had lower social index than the control-saline mice. The syn-NICD-shRNA-METH group of mice reversed the decreased social index [main effect of AAV, F _(1, 26)_ = 1.00, *P* > 0.05; METH, F _(1, 26)_ = 4.35, *P* < 0.05; AAV×METH, F _(1, 26)_ = 6.91, *P* < 0.05]. **4F.** The syn-NICD-OE-METH group of mice showed a lower social index than the control-METH group of mice [main effect of AAV, F _(1, 27)_ = 7.61, *P* < 0.05; METH, F _(1, 27)_ = 31.07, *P* < 0.001; AAV×METH, *P* > 0.05]. **4B-4C, 4G-4H.** MIP mice spent a significantly lower proportion of time in the open arms and made a lower proportion of entries into the open arms than the control saline group, indicating a higher level of anxiety in MIP mice. **4B-4C.** Syn-NICD-shRNA-METH group of mice reversed the decrease time that spent in the open arms [**4B**, main effect of AAV, F _(1, 28)_=6.60, *P* < 0.05; METH, F _(1, 28)_ = 21.19, *P* < 0.001; AAV×METH, *P*>0.05] and the number of entries into the open arms [**4C**, main effect of AAV, F _(1, 28)_ = 4.61, *P* < 0.05; METH, F _(1, 28)_ = 9.25, *P* < 0.01; AAV×METH, *P* > 0.05 ]. **4G-4H.** Syn-NICD-OE-METH group of mice aggravated the decreased time that spent in the open arms [**4G**, main effect of AAV, F _(1, 27)_ = 4.45, *P* < 0.05; METH ,F _(1, 27)_ = 31.03, *P* < 0.001; AAV×METH, *P* > 0.05] and the number of entries in the open arms [**4H**, main effect of AAV, F _(1, 24)_ = 5.15, *P* < 0.05; METH, F _(1, 24)_ = 16.67, *P* < 0.001; AAV×METH, *P* > 0.05]. **4D-4E, 4I-4J.** METH-treated mice had greater percentages of immobility time in the TST and FST than control mice. The syn-NICD-shRNA-METH group of mice reversed the increased percentage of immobility time in the TST and FST. [**4D**, main effect of AAV, F _(1, 28)_ = 7.65, *P* < 0.05; METH, F _(1, 28)_ = 7.00, *P* < 0.05; AAV×METH, *P* > 0.05; **4E**. [main effect of AAV, F _(1, 25)_ = 5.14, *P* < 0.05; METH, F _(1, 25)_ = 14.21, *P*<0.01; AAV×METH, *P* > 0.05]. **4I-4J.** The syn-NICD-OE-METH group of mice exhibited an increased percentage of immobility time in the TST and FST [**4I**, main effect of AAV, F _(1, 26)_ = 4.92, *P* < 0.05; METH, F _(1, 26)_ = 4.19, *P* = 0.051; AAV×METH, *P* > 0.05; **4J.** [main effect of AAV, F _(1, 24)_ = 6.31, *P* < 0.05; METH, F _(1, 24)_ = 24.52, *P* < 0.001; AAV×METH, *P* > 0.05]. **P* < 0.05, ***P* < 0.01 ****P* < 0.001 vs. saline control group. # *P* < 0.05, ### *P* < 0.001 vs. the METH control group. Data were presented as mean ± S.E.M, n = 7-8.


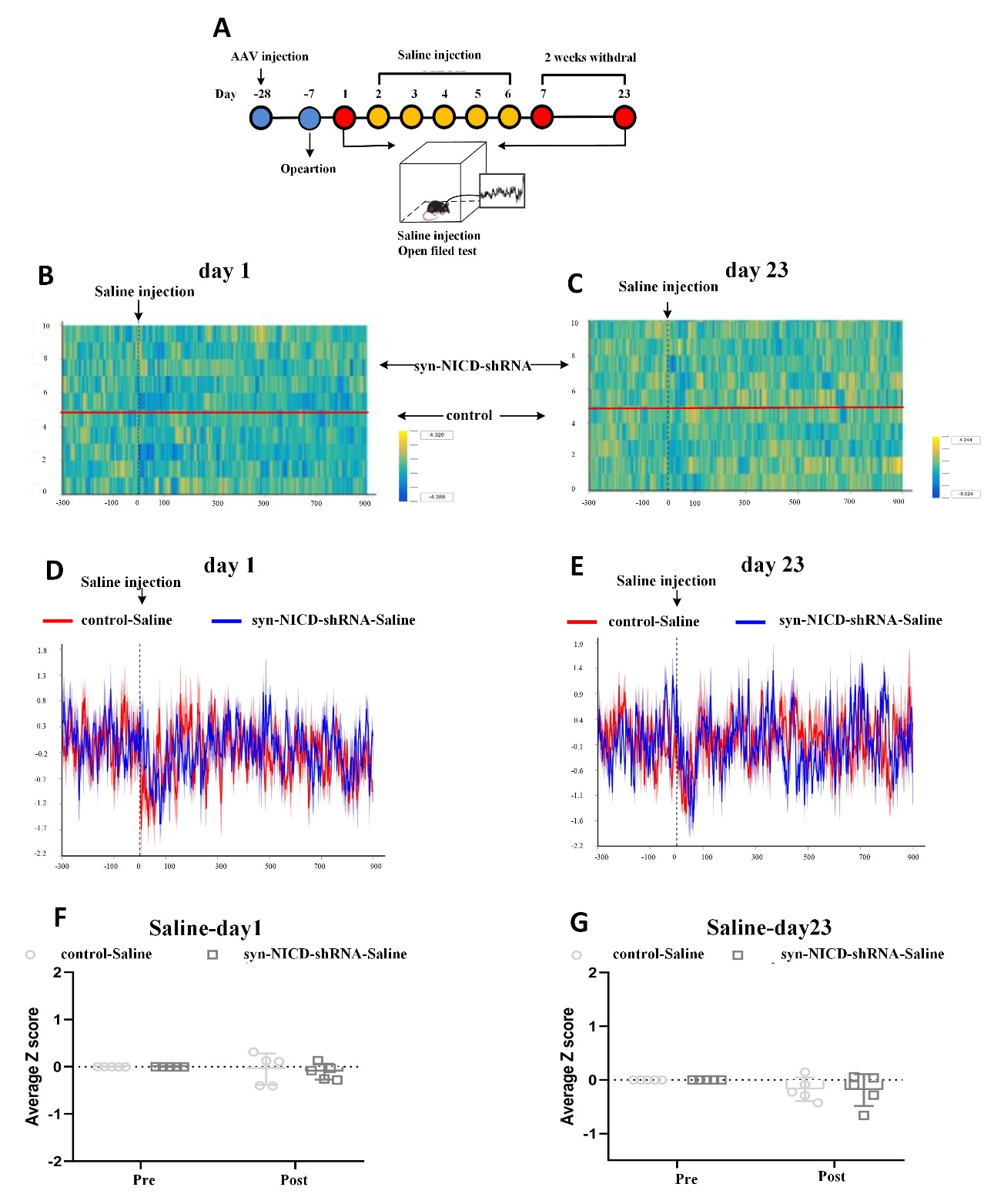


**Supplemental Figure 5. Inhibition of the Notch1 signalling did not influence the mPFC neuronal activity after saline injection. 5A.** The experimental design for recording GCaMP activity from mPFC neurons in the syn-NICD-shRNA and control groups of mice after saline injection. **5B-5C.** Heatmap illustration of Ca^2+^ signals aligned to the initiation of trials on day 1 and day 23. Each row plots one trial, and a total of 10 trials are illustrated. The colour scale on the right indicates Z scores. **5D-5E.** Average traces of calcium signals from the control mPFC neurons (red line) and the inhibitory NICD mPFC neurons (blue line) were recorded before (5 minutes) and after saline injection (15 minutes) on days 1 and 23. **5F-5G.** The average Z scores from (F) and (G) in the open field before (pre) and after (post) saline injection on day 1 and day 23. There was no significant difference between the syn-NICD-shRNA-saline group and the control-saline group on day 1 and day 23. Traces represent mean ± SEM (5D, 5E). Error bars represent mean ± SEM (5F, 5G), n = 5.


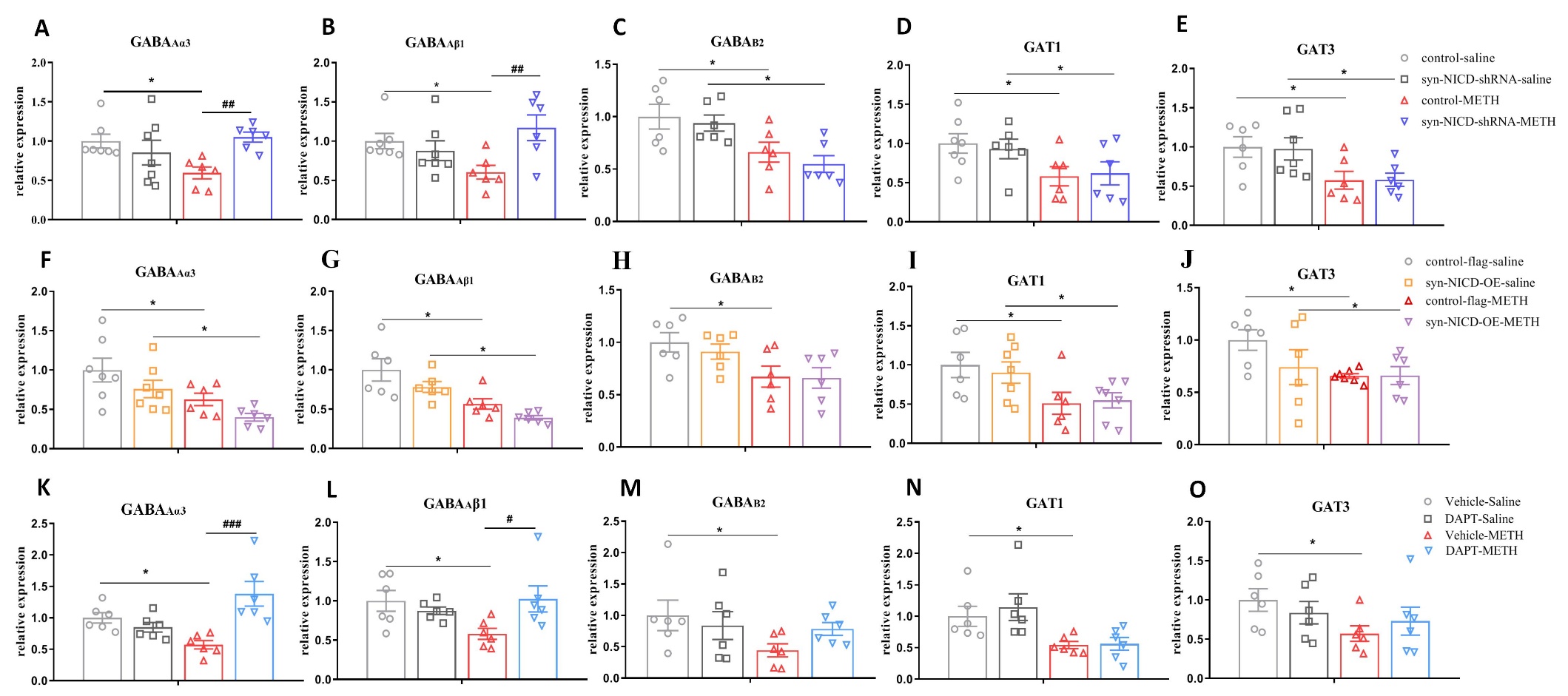


**Supplemental Figure 6. Changes in the expression of GABA receptors and transporters in the mPFC with the manipulation of mPFC Noch1 signalling in MIP mice.**

**6A-6E.** Changes in the expression of the GABAergic system in the syn-NICD-shRNA-saline and METH groups of mice. Two-way ANOVA followed LSD multiple comparisons. METH caused a significant decrease in GABA_Aα3_, GABA_Aβ1_ GABA_B2_, GAT1 and GAT3. The syn-NICD-shRNA-METH group only exhibited upregulation of GABA_Aα3_ and GABA_Aβ1_ receptor expression compared with the control METH group. **6A.** GABA_Aα3_, the main effect of AAV, F _(1, 22)_ = 2.08, *P* > 0.05; METH, F _(1, 22)_ = 0.94, *P* > 0.05; interaction, F _(1, 22)_ = 7.83, *P* < 0.05. **6B.** GABA_Aβ1_, the main effect of AAV, F _(1, 22)_ = 3.31, *P* > 0.05; METH, F _(1, 22)_ = 0.18, *P* > 0.05; interaction F _(1, 22)_ = 8.00, *P* < 0.05. **6C.** GABA_B2_, the main effect of AAV, F _(1, 20)_ = 0.87, *P* > 0.05; METH, F _(1, 20)_ = 14.97, *P* < 0.01; interaction, F _(1, 20)_ = 0.08, *P* > 0.05. **6D.** GAT1, the main effect of AAV, F _(1, 21)_ = 0.02, *P* > 0.05; METH, F _(1, 21)_ = 7.88, *P* < 0.05; interaction, F _(1, 21)_ = 0.17, *P* > 0.05. **6E.** GAT3, the main effect of AAV, F _(1, 23)_ = 0.02, *P* > 0.05; METH, F _(1, 23)_ = 11.33, *P* < 0.01; interaction, F _(1, 23)_ = 0.004, *P* > 0.05. **6F-6J.** Changes in the expression of the GABAergic system in the syn-NICD-OE-saline and METH groups of mice. There were no significant changes between syn-NICD-OE-METH and control-flag-METH group on these genes. **6F.** GABA_Aα3_, the main effect of AAV, F _(1, 22)_ = 4.47, *P* < 0.05; METH, F _(1, 22)_ =11.07, *P* < 0.05; interaction, F _(1, 22)_ = 0.004, *P* > 0.05. **6G.** GABA_Aβ1_, the main effect of AAV, F _(1, 21)_ = 6.10, *P* < 0.05; METH, F _(1, 21)_ = 21.98, *P* < 0.05; interaction, F _(1, 21)_ = 0.46, *P* > 0.05. **6H.** GABA_B2_, the main effect of AAV, F _(1, 20)_ = 0.31, *P* > 0.05; METH, F _(1, 20)_ = 10.03, *P* < 0.01; interaction, F _(1, 20)_ = 0.18, *P* > 0.05. **6I.** GAT1, the main effect of AAV, F _(1, 22)_ = 0.05, *P* > 0.05; METH, F _(1, 22)_ = 10.01, *P* < 0.01; interaction, F _(1, 22)_ = 0.25, *P* > 0.05. **6J.** GAT3, the main effect of AAV, F _(1, 21)_ = 1.55, *P* > 0.05; METH, F _(1, 21)_ = 4.50, *P* = 0.05; interaction, F _(1, 23)_ = 1.65, P > 0.05. **6K-6O.** The GABAergic system expression changes following administration of DAPT in saline and METH group of mice. The DAPT-METH only show upregulation of GABA_Aα3_ and GABA_Aβ1_ receptor expression compared with the Vehicle-METH group. **6K.** GABA_Aα3_, the main effect of DAPT, F _(1, 20)_ = 7.91, *P* < 0.05; METH, F _(1,20)_ = 0.20, *P* > 0.05; interaction, F _(1, 20)_ = 16.72, *P* < 0.001; **6L**. GABA_Aβ1_, the main effect of DAPT, F _(1, 20)_ = 1.36, *P* > 0.05; METH, F _(1, 20)_ = 1.95, *P* > 0.05; interaction, F _(1, 20)_ = 6.22, *P* < 0.05; **6M.** GABA_B2_, the main effect of DAPT, F _(1, 20)_ = 0.25, *P* > 0.05; METH, F _(1, 20)_ = 2.88, *P* > 0.05; interaction, F _(1, 20)_ = 1.95, *P* > 0.05. **6N.** For GAT1, the main effect of DAPT, F _(1, 23)_ = 0.32, *P* > 0.05; METH, F _(1, 20)_ = 12.89, *P* < 0.01; interaction, F _(1, 23)_ = 0.19, *P* > 0.05. **6O.** For GAT3, the main effect of DAPT, F _(1, 20)_ = 0.001, *P* > 0.05, METH, F _(1, 20)_ = 3.48, *P* > 0.05, interaction, F _(1, 23)_ = 1.26, *P* > 0.05. **P* < 0.05, compared to the paired saline group. #*P*< 0.05, ## *P*< 0.01, ###*P*<0.001 compared to the paired METH group. Data were presented as mean ± S.E.M, n = 6-7.
